# Non‐negative matrix factorisation of Raman spectra finds common patterns relating to neuromuscular disease across differing equipment configurations, preclinical models and human tissue

**DOI:** 10.1002/jrs.6480

**Published:** 2022-12-22

**Authors:** James J. P. Alix, Maria Plesia, Chlöe N. Schooling, Alexander P. Dudgeon, Catherine A. Kendall, Visakan Kadirkamanathan, Christopher J. McDermott, Gráinne S. Gorman, Robert W. Taylor, Richard J. Mead, Pamela J. Shaw, John C. Day

**Affiliations:** ^1^ Sheffield Institute for Translational Neuroscience University of Sheffield Sheffield UK; ^2^ Neuroscience Institute University of Sheffield Sheffield UK; ^3^ Department of Automatic Control and Systems Engineering University of Sheffield Sheffield UK; ^4^ Biophotonics Research Unit Gloucestershire Hospitals NHS Foundation Trust Gloucester UK; ^5^ Biomedical Spectroscopy, School of Physics and Astronomy University of Exeter Exeter UK; ^6^ Interface Analysis Centre, School of Physics University of Bristol Bristol UK; ^7^ Wellcome Centre for Mitochondrial Research, Translational and Clinical Research Institute, Faculty of Medical Sciences Newcastle University Newcastle upon Tyne UK; ^8^ NHS Highly Specialised Service for Rare Mitochondrial Disorders Newcastle upon Tyne Hospitals NHS Foundation Trust Newcastle upon Tyne UK

**Keywords:** amyotrophic lateral sclerosis, data analysis, muscular dystrophy, neuromuscular disease, non‐negative matrix factorisation

## Abstract

Raman spectroscopy shows promise as a biomarker for complex nerve and muscle (neuromuscular) diseases. To maximise its potential, several challenges remain. These include the sensitivity to different instrument configurations, translation across preclinical/human tissues and the development of multivariate analytics that can derive interpretable spectral outputs for disease identification. Nonnegative matrix factorisation (NMF) can extract features from high‐dimensional data sets and the nonnegative constraint results in physically realistic outputs. In this study, we have undertaken NMF on Raman spectra of muscle obtained from different clinical and preclinical settings. First, we obtained and combined Raman spectra from human patients with mitochondrial disease and healthy volunteers, using both a commercial microscope and in‐house fibre optic probe. NMF was applied across all data, and spectral patterns common to both equipment configurations were identified. Linear discriminant models utilising these patterns were able to accurately classify disease states (accuracy 70.2–84.5%). Next, we applied NMF to spectra obtained from the *mdx* mouse model of a Duchenne muscular dystrophy and patients with dystrophic muscle conditions. Spectral fingerprints common to mouse/human were obtained and able to accurately identify disease (accuracy 79.5–98.8%). We conclude that NMF can be used to analyse Raman data across different equipment configurations and the preclinical/clinical divide. Thus, the application of NMF decomposition methods could enhance the potential of Raman spectroscopy for the study of fatal neuromuscular diseases.

## Graphical abstract

Spectra from different settings were pooled and subjected to non‐negative matrix factorisation. The spectral patterns derived were used to identify disease in each paradigm. Classification performance was superior to traditional principal component‐based analysis.

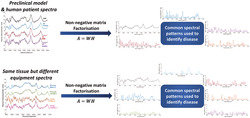

## 1 INTRODUCTION

Neuromuscular diseases are a group of neurological conditions that cause progressive weakness, resulting in significant morbidity and in many cases death. Examples include amyotrophic lateral sclerosis, mitochondrial disease and Duchenne muscular dystrophy (DMD). A variety of different investigations are used to assess patients for these conditions, including electromyography [1], imaging (e.g. ultrasound and MRI) [2], muscle biopsy [3] and genetic testing [4]. Each test has its own advantages/disadvantages; for example, with electromyography, multiple muscles can be sampled, but findings can lack specificity, whereas muscle biopsy tends to have more limited sampling but can provide a specific molecular diagnosis. Although useful in identifying disease, many of these investigations are not particularly suitable for monitoring disease or are still under development for this purpose. With increasing numbers of treatments being evaluated in trials, there is a pressing need across the neuromuscular disease spectrum for translational biomarkers that can objectively monitor symptom progression. Furthermore, quantitative readouts of disease state that cross the preclinical/clinical divide and provide a translational readout would help pull‐through promising therapeutic candidates into clinical trials.

Raman spectroscopy is a candidate biomarker for complex neuromuscular diseases. We have recently demonstrated spontaneous Raman spectroscopy of muscle as a biomarker of muscle health through both in vivo preclinical recordings [5] and ex vivo studies of human tissue [6, 7]. The simplicity of the technique, which requires no sample preparation and is quick to perform, is in stark contrast to the complex and time‐consuming assays usually required to provide biochemical information on clinical samples. Complementary to our studies on muscle is a growing body of evidence on the potential of Raman spectroscopy to analyse biofluids and tissue specimens across a range of neurological disorders such as amyotrophic lateral sclerosis [8, 9, 10], dementia [11] and Huntington's disease [12].

As medical applications of Raman spectroscopy have expanded, so too has an appreciation of the challenges required to implement Raman in the study of human disease. A variety of equipment configurations are used to study biomedical specimens, and the generation of cross‐platform data models has been identified as key development need [13]. In addition, a wide variety of analytical techniques have been applied to Raman data, including different preprocessing methods, feature selection/extraction approaches and class modelling algorithms [14]. Significant progress has been made in developing a common understanding of the advantages/disadvantages of different chemometric approaches, which can in turn drive real world applications [15, 16].

Statistical modelling of Raman data typically begins with dimension reduction, which makes data visualisation and further computational processes easier. Although a variety of different techniques can be applied to Raman spectra, principal component analysis (PCA) is the most widely used [15, 16]. In PCA, preprocessed Raman spectra are decomposed into a number of orthogonal components (principal components) which are presented in decreasing order of explained variance. PCA is easy to implement, unsupervised and requires only limited user input, which is largely restricted to defining the components taken into classification models. These can be selected according to, for example, the amount of total variance explained across a number of components (e.g. 90% of all variance), or a threshold for individual component contributions to total variance. Disadvantages of PCA relate to the interpretability of the output components, which can cancel out and have negative values, resulting in physically unrealistic representations of Raman spectra.

We have recently explored non‐negative factorisation techniques with spectral data [17, 18]. Non‐negative matrix factorisation (NMF) produces a parts‐based representation of the original data, in which two smaller matrices contain, in the context of Raman spectroscopy, the dominant spectral patterns and their relative importance to each of the original spectra [19, 20]. The non‐negative constraint results in a realistic representation of the data [17] and a further advantage is that, as an unsupervised technique, the factorisation will find dominant patterns across data without influence by class groupings. Raman data from different equipment configurations and tissue types can therefore be combined in a single factorisation, allowing simultaneous analysis across the combined data set.

We hypothesised that NMF would be able to find disease‐relevant spectral patterns across different equipment configurations and data obtained from both preclinical models and human patients. To test this, we applied NMF to human muscle spectra collected using a commercial Raman microscope and in‐house fibre optic probe, using samples taken from patients with mitochondrial disease and healthy volunteers. Next, we utilised NMF on fibre optic probe spectra obtained from the *mdx* mouse model of DMD, as well as human muscular dystrophy conditions. We found that NMF identified common spectral fingerprints across these paradigms and thus might aid the development of Raman spectroscopy as a methodology for the assessment of neuromuscular diseases.

## 2 METHODS

### 2.1 Fibre optic and microscope Raman spectroscopy

The fibre optic probe comprises a 0.5‐mm fibre optic Raman probe within a standard 21‐guage hypodermic needle, coupled to an 830‐nm semiconductor laser (Innovative Photonics Solutions) [21]. Identical low‐OH fibres (Thorlabs, Inc.) were used for delivery and collection of light. For removal of inelastically scattered light and fibre‐related fluorescence within the delivery path, in‐line laser wavelength bandpass filters were deployed (Semrock Inc USA.). A long pass filter removed elastically scattered light in the return path. The collecting fibre was optically coupled to the spectrometer (Raman Explorer Spectrograph, Headwall Photonics, Inc. and iDus 420BR‐DD CCD camera, Andor Technology, Ltd.). Laser power was 60 mW at the distal tip of the probe.

Microscope Raman spectra were collected using a ×50 objective, 830‐nm excitation laser, coupled with a Renishaw Raman spectrometer system (System 1000, Renishaw Plc.). Laser power was 30 mW at the objective. Acquisition time at each site for both the probe and microscope was 40 s.

### 2.2 Preclinical studies

Male mice (*n* = 8) of the C57/Bl10 *mdx* model of DMD were used. As all mice in the colony carry the Dmdmdx allele (breeding utilised homozygous female mice and hemizygous males), wild‐type male C57BL/10ScSnOlaHsd (C57Bl/10) mice (*n* = 8) were used as a healthy control (purchased from Envigo). Mouse breeding was undertaken in a specified pathogen‐free environment. Experimental work was undertaken in a standard preclinical facility (12‐h light/dark cycle and room temperature 21°C). All procedures were undertaken with the approval of the University of Sheffield Ethical Review Sub‐Committee and UK Home Office (licence number 70/8587), in accordance with the Animal (Scientific Procedures) Act 1986. The ARRIVE guidelines were followed [22]. In vivo fibre optic Raman spectroscopy was undertaken as previously described [5]. Briefly, mice were anaesthetised, hindlimb fur removed and the fibre optic Raman probe inserted into both the medial and lateral heads of both gastrocnemius muscles.

### 2.3 Human tissue

Muscle tissue from a total of 20 patients with neuromuscular conditions was collected through either conchotome needle biopsy, open muscle biopsy, or at the time of surgery and snap frozen. These included *n* = 14 patients with genetically confirmed mitochondrial disease (*n* = 11 with m.3243A > G mutation, *n* = 3 POLG‐related, *n* = 1 single large‐scale DNA mutation; see Table S1 and Alix et al. [6] for further details). Tissue was also obtained from patients diagnosed with dystrophic myopathy (*n* = 3 limb girdle muscular dystrophy and *n* = 1 desmin myopathy; see Table S2). In addition, two samples were obtained from patients with DMD (at the time of spinal surgery; Table S3). Lastly, muscle tissue was taken from *n* = 10 healthy volunteers with no known neurological illness at the time of surgery for anterior cruciate repair (see Table S1 for further details). Each participant contributed one sample to the analysis. The use of human tissue was approved by NHS Research Ethics Committees (references 16/YH/0261 and 09/H0906/75). For the microscope/fibre optic probe factorisation, basic details for the human participants detailed in Section 3.1 of the main manuscript are found in Table S1. For the *mdx* mouse/human factorisation (Section 3.2), we utilised samples from healthy volunteers and adult patients with dystrophic muscle conditions (Table S2). This is because we were only able to obtain two DMD samples from children but had access to four adult dystrophic myopathy samples which will share some common pathological features with the mice (e.g. necrotic fibres and regenerating fibres). Furthermore, we did not have access to age‐matched healthy tissue from children.

Prior to experiments, all samples were stored at −80°C. For Raman data collection, samples were thawed to room temperature and placed on a calcium fluoride slide. Spectra were first collected from the fibre optic probe and then transferred to the microscope. With both equipment formats, spectra were obtained from two to six sites on each sample. Similar regions of each sample were chosen for examination by both the microscope and probe, accepting that this only defined a region for spectral acquisition, rather matching the exact location across both equipment formats.

### 2.4 Data analysis

Analysis was performed using custom codes in MATLAB (MATLAB R2021b The MathWorks, Inc., Natick, MA). For probe versus microscope comparisons, spectra were first windowed to 900–1700 cm^−1^; for probe‐only mouse versus human sample comparisons, spectra were windowed to 900–1800 cm^−1^. The reason for removing below 900 cm^−1^ was that the spectra obtained from the fibre optic probe are dominated by silica artefact below this wavenumber. For all analyses, interpolation to integer wavenumber spacing was undertaken, followed by background subtraction using the adaptive, iteratively reweighted penalised least squares algorithm [23], Savitzky–Golay smoothing (second order, frame length 5) and standard normal variate normalisation. As the latter produces spectra with an arbitrary negative intensity, the minimum spectral intensity was then added to all spectra to remove the negativity.

Hierarchical alternating least squares NMF factorisation was performed [24]. In order to ensure solution stability, a non‐negative singular value decomposition, low rank correction algorithm was used [25]. NMF approximates the data set of an n × m matrix, **A** (n samples of length m), as the product of two low‐rank matrixes (**W** and **H**):

A=WH,

where for an r‐rank factorisation, ***W*** is size n × r and ***H*** is size r × m. The rank matrix, ***H***, contains r modes, which are the dominant spectral patterns within the original data. In the weighting matrix, ***W***, each sample is assigned a weight corresponding to each spectral pattern, which denotes the importance of a given pattern to that sample.

To select the number of spectral patterns (or rank, r) to be generated in the factorisation, recordings from wild‐type mice were used. This was done by separating right leg and left recordings into two matrices, **L** and **R**. The root mean square residual between these sides is calculated as

$$N=\sqrt{\frac{\sum_{i=1}^{n}\sum_{j=1}^{m}{\left|R_{\mathrm{ij}}-L_{\mathrm{ij}}\right|}^{2}}{n\times m}\;}.$$

Because no biological difference between the two legs is anticipated, *N* represents the biological noise within the data. For each reconstruction, the root mean square residual between the data set (***A***) and the approximation (***WH***) was also determined:

$$D=\sqrt{\frac{\sum_{i=1}^{n}\sum_{j=1}^{m}{\left|A_{\mathrm{ij}}-{\left(\mathrm{WH}\right)}_{\mathrm{ij}}\right|}^{2}}{n\times m}\;}.$$

The solution which exceeded the approximation of left leg/right leg (i.e. when *D* < *N*) gave the chosen rank. Comparisons of spectral weightings between disease/healthy groups were then undertaken using nested *t*‐tests (spectra nested within samples, GraphPad Prism, version 9). Statistical significance was taken as *p* < 0.05.

Classification models were generated using linear discriminant analysis (LDA) models. First, all modes were input into the classifier, and a leave‐three‐out cross‐validation was performed (cv), in which data from three samples or mice (not spectra) were left out. Different combinations of three left out across 500 iterations without replacement, i.e. each specific combination of three was used only once. For both human and mouse data, cv was first done on an individual sample/mouse basis. For this, spectral weights from each spectrum taken from a given sample/mouse were averaged, so that the sample/mouse input a single weight into the classifier. In addition, cv was repeated on an individual spectral basis, in which all spectra from a given sample were left out, and all remaining spectra classified individually. For comparison, PCA‐fed LDA (PCA‐LDA) was also performed using the same cv approaches. The number of components covering 90% of the variance of the data was chosen for input into the LDA. Accuracy, sensitivity, specificity and the area under the receiver operating characteristic curve (AUROC) were calculated.

## 3 RESULTS AND DISCUSSION

### 3.1 Same tissues: Different equipment formats

**FIGURE 1:**
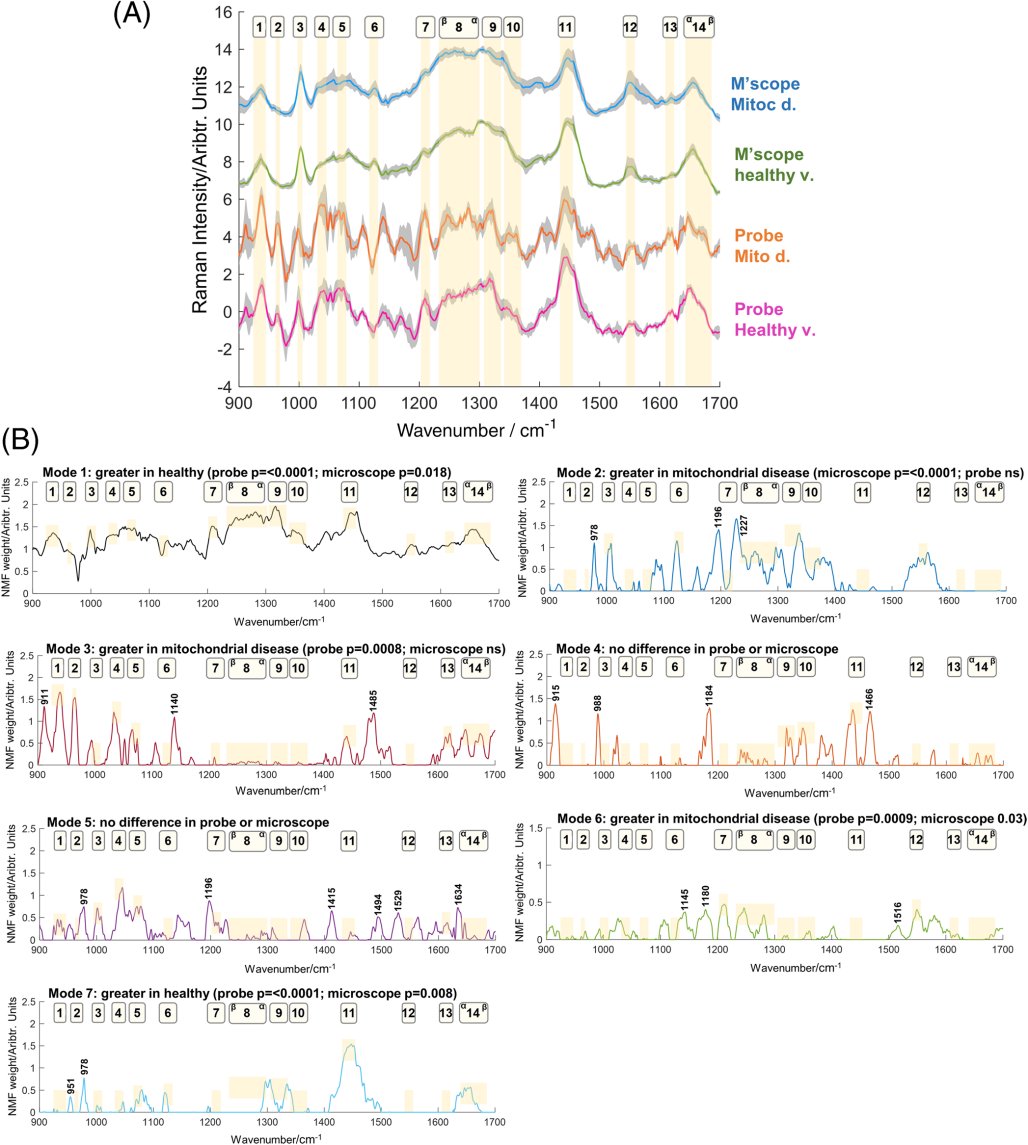
Mitochondrial disease patients and healthy volunteers: average spectra and NMF modes from microscope and probe formats. (A) Average spectra (with standard deviation shaded) from the two equipment formats. Wavenumber regions with prominent peaks are highlighted. (B) Modes from the NMF analysis. Modes 1, 6 and 7 demonstrated significant differences between patients and healthy volunteers in the same direction for both probe and microscope. Modes 2 and 3 detect patterns specific to microscope and probe formats, respectively. New peaks of interest are denoted with wavenumber labels. Mitoc., mitochondrial; M'scope, microscope; HV, healthy volunteers; NMF, non‐negative matrix factorisation; ns, nonsignificant [Colour figure can be viewed at wileyonlinelibrary.com]

Raman spectra were collected from muscle samples obtained from patients with mitochondrial disease (*n* = 14) and healthy volunteers (*n* = 10) using two different equipment formats (microscope and fibre optic probe). The spectra obtained with both the microscope and fibre optic probe demonstrated similar core features, such as peaks at 1000 cm^−1^ (phenylalanine), 1445 cm^−1^ (CH modes [CH_2_ and CH_3_ deformations: bending and scissoring] in proteins/lipids), 1550 cm^−1^ (tryptophan, proteins) and 1656 cm^−1^ (amide I, proteins) (Figure 1A). In addition, differences were also apparent, particularly between 1200 and 1350 cm^−1^ (including peaks relating to tyrosine, phenylalanine and the CH_2_CH_3_ deformation, proteins/lipids), perhaps relating to differences in power density and collection optics [6]. See Table S4 for further tentative peak assignments and references relating to average spectra.

NMF with a rank of 7 was applied across data from both formats, and the spectral weightings were compared (Figure 1B). Tentative peak assignments for new spectral features arising outside the average spectra windows are given in Table S5. Nested graphs showing between group (mitochondrial disease vs. healthy volunteers) differences are shown in Figure S1. Mode 1 demonstrated a significant difference between the mitochondrial disease and healthy groups for both the microscope (*p* = 0.02) and probe (*p* < 0.0001), with this mode increasing in healthy volunteers in both formats. Prominent peaks relating to α‐helical protein content were seen (935, 1315, 1356, and 1656 cm^−1^). α‐helices are the dominant secondary protein structure in muscle [20], and the relative prominence of these in healthy muscle suggests a transition to β‐sheet structures in myopathy [21]. In previous analyses of microscope and fibre optic probe data, we observed a relative loss of α‐helix‐related peaks in both formats [6] and so it is reassuring that this is evident in the combined factorisation. Mode 7 was also significantly more prominent in healthy volunteers across both equipment formats (microscope *p* = 0.008; probe *p* < 0.0001) and shared some similar features to mode 1. Mode 6 was significantly greater in mitochondrial disease (microscope *p* = 0.03; probe *p* = 0.0009) with prominent peaks relating to nucleotides (1180 cm^−1^), tyrosine/phenylalanine (1210 cm^−1^) and amide III random coil/β‐sheet configurations (1245 cm^−1^). Modes 2 and 3 found patterns relatively specific for the microscope and probe, respectively, with, for example, the aforementioned 1200–1350 cm^−1^ region differing between the two.

We next used linear discriminant models to further assess the ability of NMF outputs to identify disease (Table 1). Analyses utilising either all modes or only those which shared common patterns across the two equipment formats (modes 1, 6 and 7) demonstrated high classification performances. A comparison to PCA‐LDA is also shown, with NMF‐LDA demonstrating superior performance. Classification performance at the level of individual spectra yielded similar results (Table S6).

**TABLE 1:** Classification performance using all modes and only modes with common differences in both the microscope and probe.

All modes				
	Accuracy	Sensitivity	Specificity	AUROC
Microscope				
NMF‐LDA	86.4% (4.1)	77.1% (6.6)	99.2% (2.6)	0.94 (0.03)
PCA‐LDA (15 PCs)	59.1% (7.3)	62.2% (9.6)	54.6% (10.7)	0.63 (0.09)
Probe				
NMF‐LDA	81.0% (3.7)	88.5% (5.5)	70.5% (5.2)	0.77 (0.05)
PCA‐LDA (15 PCs)	57.6% (6.9)	58.0% (8.6)	57.1% (11.3)	0.50 (0.07)
Modes with common differences in both probe and microscope				
Microscope				
NMF‐LDA (1, 6 and 7)	70.5% (3.0)	66.0% (3.3)	76.9% (4.9)	0.80 (0.04)
Probe				
NMF‐LDA (1, 6 and 7)	84.3% (1.9)	87.5% (3.2)	80.0% (0.4)	0.85 (0.02)

*Note*: For comparison, results achieved by PCA‐LDA are also provided. Mean (standard deviation) are shown.

Abbreviations: AUROC, area under the receiver operating characteristic curve; LDA, linear discriminant analysis; NMF, non‐negative matrix factorisation; PCs, principal components; PCA, principal component analysis.

### 3.2 Human and mouse tissue: Same fibre optic equipment

**FIGURE 2:**
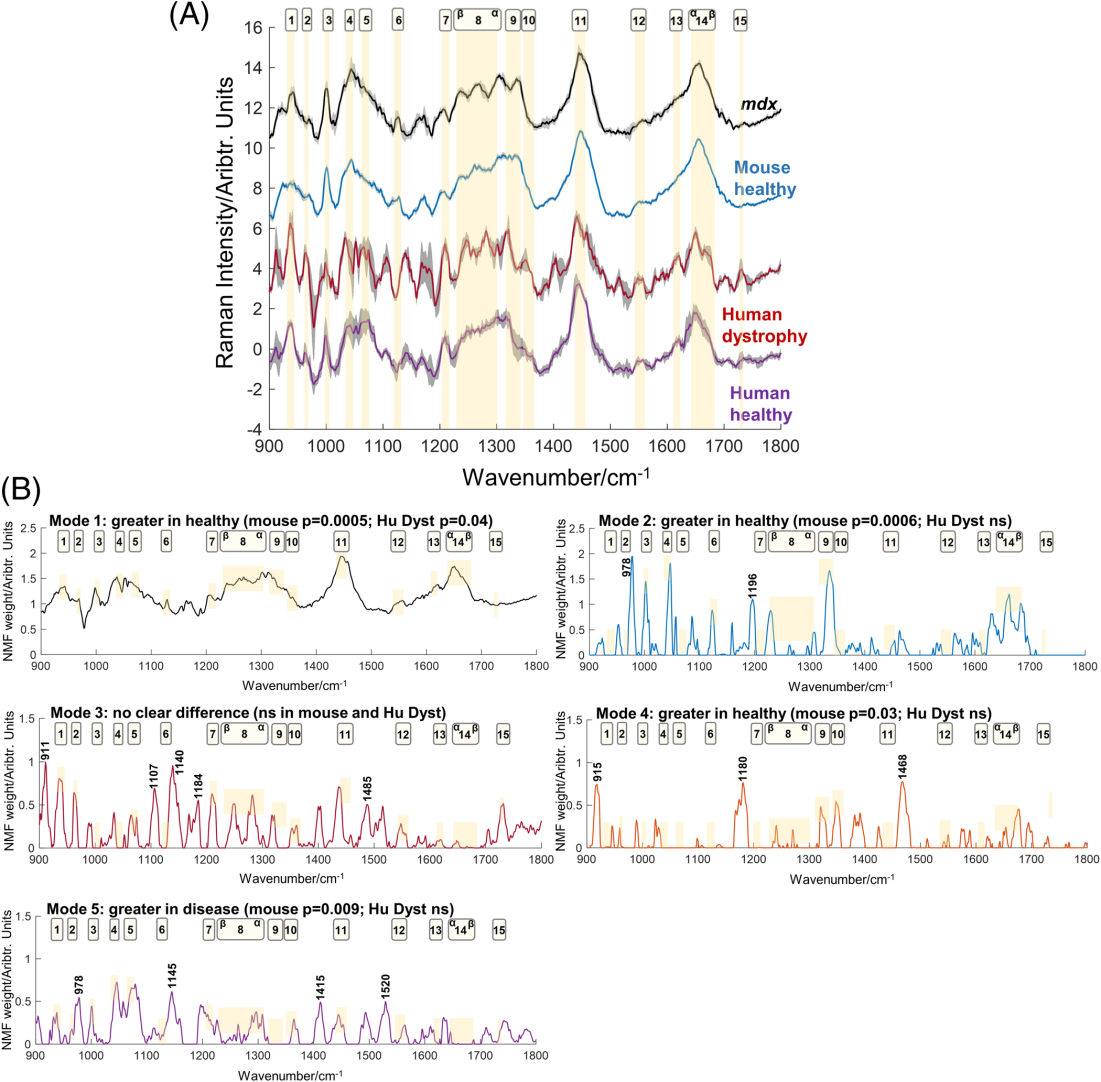
Dystrophic mouse and human muscle: average spectra and NMF modes from a fibre optic Raman probe. (A) Average spectra (with standard deviation) from mouse and human tissue. Wavenumber regions with prominent peaks are highlighted. (B) Modes from the NMF analysis. Mode 1 is significantly different in both the mouse and human comparisons in the same direction. Modes 2 is significantly different for *mdx* with the human samples trending in the same direction but not reaching statistical significance. NMF, non‐negative matrix factorisation [Colour figure can be viewed at wileyonlinelibrary.com]

Raman spectra were acquired from muscle samples obtained from patients with dystrophic muscle disease and a preclinical model of DMD, the *mdx* mouse. Prominent peaks at 1000 cm^−1^ (phenylalanine), 1450 cm^−1^ (CH modes, CH_2_ and CH_3_ deformations: bending and scissoring in proteins/lipids) and 1655 cm^−1^ were present in both mouse and human spectra (Figure 2A). In the 1250–1350 cm^−1^ region, more prominent features were evident in the *mdx* and human dystrophy samples, than in their respective healthy controls.

NMF with a rank of 5 was applied (Figure 2B; nested plots of spectral weights are shown in Figure S2). Mode 1 demonstrated dystrophy (*mdx* or human) versus healthy differences in both the mouse and human data (*mdx p* = 0.0005; human *p* = 0.04), with greater prominence of this mode in healthy muscle. Peaks associated with α‐helical structures were seen. A similar trend was also seen for mode 2, although this did not reach statistical significance in the human group (human *p* = 0.08; *mdx p* = 0.0006). Prominent features in this mode included 978 cm^−1^ (phospholipids), 1047 cm^−1^ (proteins), 1190 cm^−1^ (proline/valine), 1196 cm^−1^ (cytosine) and 1335 cm^−1^ (CH_2_CH_3_ deformation, proteins/lipids). Further healthy versus disease differences were evident in the *mdx* data in modes 4 and 5.

The balance between α‐helix and β‐sheet structures appears to be a robust biomarker of muscle health, noted not only in the preceding microscope/probe analysis on human tissue but also in our previous work in mice [5], human samples using more standard PCA feature extraction [6, 7] and the work of Gautam et al. in fly models of myopathy [26]. Biomarkers of disease that cross the preclinical/clinical divide and can provide an equivalent readout of disease state in both settings are a priority area in fatal neuromuscular conditions [27, 28, 29]. Our previous in vivo work in mice demonstrated no post‐Raman functional muscle impairment or tissue injury [5], and thus, successful in vivo human muscle recording could provide a quantitative, translational measure of muscle health suitable for preclinical and clinical studies.

As only a small number of human muscle samples were available, we did not perform multivariate modelling with the human data. Classification performance data for the *mdx* mice are shown using all modes and only modes 1 and 2 (Table 2). NMF‐LDA again outperformed PCA‐LDA. Spectral‐level classification results were similar (Table S7). It is worth noting that classification performances as high as this raise the possibility of overfitting of the data. Ideally, we would have had separate, unseen, data to validate as a test set (both in this and the preceding analysis). In the absence of this, cross‐validation is a standard approach to reduce overfitting, although we accept that the lack of dedicated test data is a weakness that could be addressed in future, larger studies.

**TABLE 2:** Classification performance for *mdx* mice using all modes and only modes that have common differences across mouse and human data.

	Accuracy	Sensitivity	Specificity	AUROC
All modes				
NMF‐LDA	98.8% (2.9)	98% (5.2)	99.7% (1.4)	0.99 (0.01)
PCA‐LDA (15 PCs)	90.8% (5.4)	82.9% (10.1)	99.8% (1.5)	0.91 (0.05)
Modes with common differences in mouse and human				
NMF‐LDA (modes 1 and 2)	98% (4.7)	96.3% (8.8)	100% (0)	0.98 (0.05)

Abbreviations: LDA, linear discriminant analysis; NMF, non‐negative matrix factorisation; PCA, principal component analysis.

NMF has been shown to effectively reduce high‐dimensional data and capture important features in different areas of medical research, including Raman spectroscopy [30]. The non‐negative constraint is particularly useful for facilitating the interpretation of latent factors within spectral data. The majority of NMF methods are iterative and converge to a local minima; however, the initialisation of the algorithm is important in determining the outputs, and random initialisation can influence the convergence and stability of the final solution [31]. Herein, we used a method shown to generate sparse initial factors [25], although other approaches are available [31]. One of the advantages of the NMF technique not explored in the present work is that once a particularly important spectral pattern (or mode) is found, this can be used for initialisation of new data. Alternatively, only a given pattern can be sought within new data. Thus, NMF may be particularly suitable for identifying and then utilising disease‐specific spectral patterns.

The variable equipment configurations used in biomedical applications of Raman spectroscopy are seen as a potential barrier to clinical translation. Although our systems underwent matching calibration, recent work has demonstrated that this does not overcome the differences between equipment platforms [13]. Average spectra from the microscope and probe demonstrated many similarities but also clear differences. Such differences are not unexpected given the differences between the microscope and probe in, for example, sampling volume, resolution and power density. In addition, although spectra were windowed to exclude silica‐related artefact, the fibres may still make a small contribution to the overall spectral profile. To facilitate the transfer of data between equipment, computational solutions have been called for, and model transfer techniques, in which a data model constructed using one set of conditions (or equipment) is used to predict new data acquired with under a different set of conditions, have been explored [32, 33]. Our approach here differs as all data were pooled, rather than obtained and analysed separately. Although we do not propose NMF decomposition as a single solution for this complex problem, our data suggest that it may provide a useful analytical framework. We would anticipate that application of NMF to equipment sets more similar in configuration, such as two microscopes or two fibre optic probes, would likely be equally, if not more, successful in identifying key disease‐related spectral profiles.

A limitation of our present work is the small number of samples available, particularly the paucity of DMD muscle tissue for the *mdx/*human modelling. The human diseases studied are all considered rare, and muscle tissue can only be obtained with an invasive biopsy. Acquiring such tissue can therefore be challenging, particularly in children. Although the adult dystrophy samples will share some common pathological features to the *mdx* mouse, a comparison with DMD tissue would have been preferable. During the study, we did obtain two DMD muscle samples; however, the lack of age‐matched healthy muscle tissue adds an additional uncertainty to the interpretation of the NMF outputs. We did attempt analysis using DMD tissue together with our youngest healthy volunteer tissue (see Table S3) and achieved similar results to the adult human dystrophy analysis (Figure S3 and Table S8). Thus, a study utilising a larger number of samples and/or more closely matched human/mouse samples would be useful. The characteristic spectral patterns and disease classification performance may, of course, change in such a study. A small study such as ours does, however, provide early data that can be used to calculate sample sizes in future studies and help ensure the scientific (and ethical) validity of more costly studies. Encouragingly, previous Raman works that have moved from small sample size, proof of concept stages to larger testing have generally demonstrated preserved diagnostic performance [34, 35]. Recent proposals on data sharing to generate large databases for modelling purposes may be also useful in testing how robust NMF‐based decompositions are in disease detection [13].

## 4 CONCLUSIONS

In this paper, we have shown that NMF can be used to extract common spectral patterns in data acquired from different equipment formats and human/mouse muscle. The NMF outputs were able to classify disease with greater accuracy than standard PCA‐LDA models. NMF decomposition methods may help combine data in multicentre studies and facilitate the translation of preclinical work to human patients.

## Supporting information


**Figure S1.** Nested plots of NMF weight for each spectrum in the probe microscope analysis.

**Figure S2.** Nested plots of NMF weight for each spectrum in the *mdx/*human muscular dystrophy analysis.

**Figure S3.** Average spectra and NMF modes for mdx, and human DMD analyses

**Table S1.** Demographic and clinical details for the human participants used in the microscope/fibre optic probe study.
**Table S2.** Demographic and clinical details for the human participants used in the mouse/human study presented in the main manuscript. Age/gender matched healthy volunteer samples were selected from those presented in supplementary Table 1.
**Table S3.** Demographic and clinical details for the human participants used in the mouse/human study presented in the supplement data. Healthy volunteer samples were selected from those presented in supplementary Table 1.
**Table S4.** Tentative peak assignments of prominent spectral features in the average spectra plots.
**Table S5.** Tentative peak assignments for additional spectral features identified within the NMF mode plots.
**Table S6.** Classification performance on individual spectra: probe/microscope – human mitochondrial disease vs. healthy volunteers.
**Table S7.** Classification performance on individual spectra in the *mdx*/human muscular dystrophy analysis.
**Table S8.** Classification performance using all modes: *mdx* vs. human DMD. For comparison, results achieved by PCA‐LDA using PCs which cover 90% of the data variance are shown.

## Data Availability

The data that support the findings of this study are available from the corresponding author upon reasonable request.

## References

[1] J. R. Daube , D. I. Rubin , Muscle Nerve 2009, 39, 244. 19145648 10.1002/mus.21180

[2] J. R. Dahlqvist , P. Widholm , O. D. Leinhard , J. Vissing , Ann.Neurol. 2020, 88, 669. 32495452 10.1002/ana.25804

[3] J. S. Nix , S. A. Moore , J. Neuropath , Exp. Neurol. 2020, 79, 719. 10.1093/jnen/nlaa046 PMC7304986 32529201

[4] R. Thompson , S. Spendiff , A. Roos , P. R. Bourque , J. Warman Chardon , J. Kirschner , R. Horvath , H. Lochmüller , Lancet Neurol. 2020, 19, 522. 32470424 10.1016/S1474-4422(20)30028-4

[5] M. Plesia , O. A. Stevens , G. R. Lloyd , C. A. Kendall , I. Coldicott , A. J. Kennerley , G. Miller , P. J. Shaw , R. J. Mead , J. C. C. Day , J. J. P. Alix , A. C. S. Chem , Neuroscience 2021, 12, 1768. 10.1021/acschemneuro.0c00794 PMC8154326 33950665

[6] J. J. P. Alix , M. Plesia , G. R. Lloyd , A. P. Dudgeon , C. A. Kendall , C. J. McDermott , G. S. Gorman , R. W. Taylor , P. J. Shaw , J. C. Day , J. Raman Spectrosc. 2022, 53, 172.

[7] J. J. P. Alix , M. Plesia , G. R. Lloyd , A. P. Dudgeon , C. A. Kendall , C. Hewamadduma , M. Hadjivassiliou , C. J. McDermott , G. S. Gorman , R. W. Taylor , P. J. Shaw , J. C. C. Day , Analyst 2022, 147, 2533. 35545877 10.1039/d1an01932e PMC9150427

[8] C. F. Morasso , D. Sproviero , M. C. Mimmi , M. Giannini , S. Gagliardi , R. Vanna , L. Diamanti , S. Bernuzzi , F. Piccotti , M. Truffi , O. Pansarasa , F. Corsi , C. Cereda , Nanomedicine 2020, 29, 102249. 32599162 10.1016/j.nano.2020.102249

[9] D. Ami , A. Duse , P. Mereghetti , F. Cozza , F. Ambrosio , E. Ponzini , R. Grandori , C. Lunetta , S. Tavazzi , F. Pezzoli , A. Natalello , Anal. Chem. 2021, 93, 16995. 34905686 10.1021/acs.analchem.1c02546 PMC8717331

[10] Q. J. Zhang , Y. Chen , X. H. Zou , W. Hu , M. L. Ye , Q. F. Guo , X. L. Lin , S. Y. Feng , N. Wang , Ann. Clin. Transl. Neurol. 2010, 2020, 7. 10.1002/acn3.51194 PMC7545607 32951348

[11] M. Paraskevaidi , C. L. M. Morais , D. E. Halliwell , D. M. A. Mann , D. Allsop , P. L. Martin‐Hirsch , F. L. Martin , A. C. S. Chem , Neuroscience 2018, 9, 2786. 10.1021/acschemneuro.8b00198 29865787

[12] A. Huefner , W. L. Kuan , S. L. Mason , S. Mahajan , R. A. Barker , Chem. Sci. 2020, 11, 525. 32190272 10.1039/c9sc03711j PMC7067270

[13] S. Guo , C. Beleites , U. Neugebauer , S. Abalde‐Cela , N. K. Afseth , F. Alsamad , S. Anand , C. Araujo‐Andrade , S. Askrabic , E. Avci , M. Baia , M. Baranska , E. Baria , L. A. E. Batista de Carvalho , P. de Bettignies , A. Bonifacio , F. Bonnier , E. M. Brauchle , H. J. Byrne , I. Chourpa , R. Cicchi , F. Cuisinier , M. Culha , M. Dahms , C. David , L. Duponchel , S. Duraipandian , S. F. El‐Mashtoly , D. I. Ellis , G. Eppe , G. Falgayrac , O. Gamulin , B. Gardner , P. Gardner , K. Gerwert , E. J. Giamarellos‐Bourboulis , S. Gizurarson , M. Gnyba , R. Goodacre , P. Grysan , O. Guntinas‐Lichius , H. Helgadottir , V. M. Grosev , C. Kendall , R. Kiselev , M. Kolbach , C. Krafft , S. Krishnamoorthy , P. Kubryck , B. Lendl , P. Loza‐Alvarez , F. M. Lyng , S. Machill , C. Malherbe , M. Marro , M. P. M. Marques , E. Matuszyk , C. F. Morasso , M. Moreau , H. Muhamadali , V. Mussi , I. Notingher , M. Z. Pacia , F. S. Pavone , G. Penel , D. Petersen , O. Piot , J. V. Rau , M. Richter , M. K. Rybarczyk , H. Salehi , K. Schenke‐Layland , S. Schlucker , M. Schosserer , K. Schutze , V. Sergo , F. Sinjab , J. Smulko , G. D. Sockalingum , C. Stiebing , N. Stone , V. Untereiner , R. Vanna , K. Wieland , J. Popp , T. Bocklitz , Anal. Chem. 2020, 92, 15745. 33225709 10.1021/acs.analchem.0c02696

[14] H. J. Butler , L. Ashton , B. Bird , G. Cinque , K. Curtis , J. Dorney , K. Esmonde‐White , N. J. Fullwood , B. Gardner , P. L. Martin‐Hirsch , M. J. Walsh , M. R. McAinsh , N. Stone , F. L. Martin , Nat. Protoc. 2016, 11, 664. 26963630 10.1038/nprot.2016.036

[15] C. L. M. Morais , K. M. G. Lima , M. Singh , F. L. Martin , Nat. Protoc. 2020, 15, 2143. 32555465 10.1038/s41596-020-0322-8

[16] S. Guo , J. Popp , T. Bocklitz , Nat. Protoc. 2021, 16, 5426. 34741152 10.1038/s41596-021-00620-3

[17] A. L. Blee , J. C. C. Day , P. E. J. Flewitt , A. Jeketo , D. Megson‐Smith , J. Raman Spectrosc. 2021, 52, 1135.

[18] C. N. Schooling , T. Jamie Healey , H. E. McDonough , S. J. French , C. J. McDermott , P. J. Shaw , V. Kadirkamanathan , J. J. P. Alix , Physiol. Meas. 2021, 42, 105004. 10.1088/1361-6579/ac2672 34521070

[19] Y. X. Wang , Y. J. Zhang , IEEE Trans. Knowl. Data Eng. 2013, 25, 1336.

[20] D. D. Lee , H. S. Seung , Nature 1999, 401, 788. 10548103 10.1038/44565

[21] J. C. Day , N. Stone , Appl. Spectrosc. 2013, 67, 349. 23452501 10.1366/12-06651

[22] C. Kilkenny , W. J. Browne , I. C. Cuthill , M. Emerson , D. G. Altman , PLoS Biol. 2010, 8, e1000412. 20613859 10.1371/journal.pbio.1000412 PMC2893951

[23] Z. M. Zhang , S. Chen , Y. Z. Liang , Analyst 2010, 135, 1138. 20419267 10.1039/b922045c

[24] A. Cichocki , R. Zdunek , S.‐I. Amari , in Independent Component Analysis and Signal Separation. ICA 2007 (Eds: M. E. Davies , C. J. James , S. A. Abdallah , M. D. Plumbley ),Lecture Notes in Computer Science, Vol. 4666, Springer, Berlin, Heidelberg 2007.

[25] S. M. Atif , S. Qazi , N. Gillis , Pattern Recognit. Lett. 2019, 122, 53.

[26] R. Gautam , S. Vanga , A. Madan , N. Gayathri , U. Nongthomba , S. Umapathy , Anal. Chem. 2015, 87, 2187. 25583313 10.1021/ac503647x

[27] M. D. Grounds , J. R. Terrill , B. A. Al‐Mshhdani , M. N. Duong , H. G. Radley‐Crabb , P. G. Arthur , Dis. Model Mech. 2020, 13, dmm043638. 32224496 10.1242/dmm.043638 PMC7063669

[28] O. M. Russell , G. S. Gorman , R. N. Lightowlers , D. M. Turnbull , Cell 2020, 181, 168. 32220313 10.1016/j.cell.2020.02.051

[29] M. C. Kiernan , S. Vucic , K. Talbot , C. J. McDermott , O. Hardiman , J. M. Shefner , A. Al‐Chalabi , W. Huynh , M. Cudkowicz , P. Talman , L. H. Van den Berg , T. Dharmadasa , P. Wicks , C. Reilly , M. R. Turner , Nat. Rev. Neurol. 2021, 17, 104. 33340024 10.1038/s41582-020-00434-z PMC7747476

[30] K. Milligan , X. Deng , P. Shreeves , R. Ali‐Adeeb , Q. Matthews , A. Brolo , J. J. Lum , J. L. Andrews , A. Jirasek , Sci. Rep. 2021, 11, 3853. 33594122 10.1038/s41598-021-83343-5 PMC7886912

[31] F. Esposito , Mathematics 2021, 9, 1006.

[32] S. Guo , R. Heinke , S. Stöckel , P. Rösch , J. Popp , T. Bocklitz , J. Raman Spectrosc. 2018, 49, 627. 10.1021/acs.analchem.8b01536 30016081

[33] S. Guo , A. Kohler , B. Zimmermann , R. Heinke , S. Stockel , P. Rosch , J. Popp , T. Bocklitz , Anal. Chem. 2018, 90, 9787. 30016081 10.1021/acs.analchem.8b01536

[34] U. Utzinger , D. L. Heintzelman , A. Mahadevan‐Jansen , A. Malpica , M. Follen , R. Richards‐Kortum , Appl. Spectrosc. 2001, 55, 955.

[35] C. Zheng , S. Qing , J. Wang , G. Lu , H. Li , X. Lu , C. Ma , J. Tang , X. Yue , Photodiagn. Photodyn. Ther. 2019, 27, 156. 10.1016/j.pdpdt.2019.05.029 31136828

