# Supplementary figures and images for: Non‐negative matrix factorisation of Raman spectra finds common patterns relating to neuromuscular disease across differing equipment configurations, preclinical models and human tissue
Source: J Raman Spectrosc. 2022 Dec 22;54(3):258–68. doi: 10.1002/jrs.6480 (PMC10947050; doi:10.1002/jrs.6480)

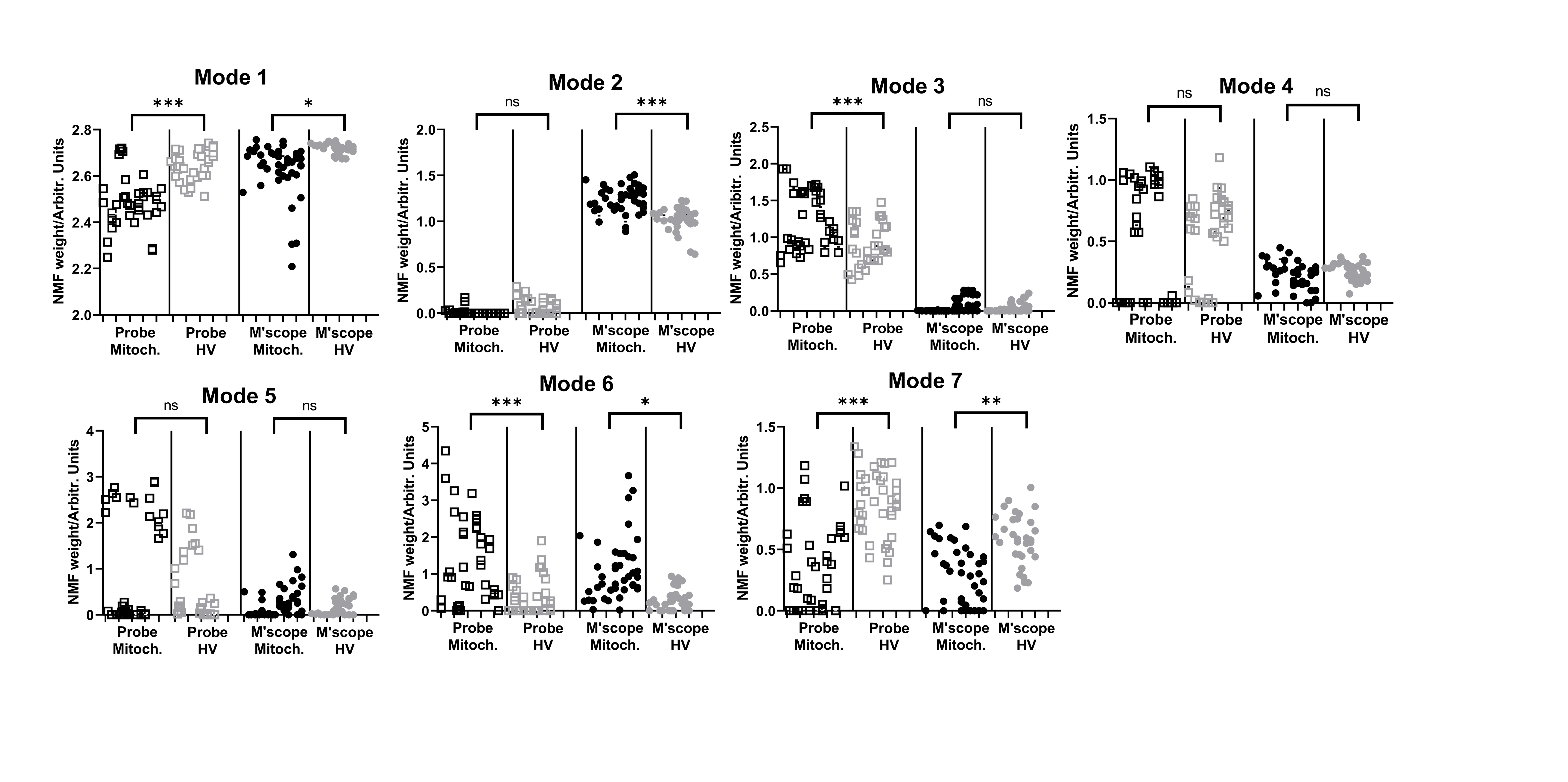

Supplement: Supplementary file 1 — Figure S1. Nested plots of NMF weight for each spectrum in the probe microscope analysis. [file JRS-54-258-s001.tiff]

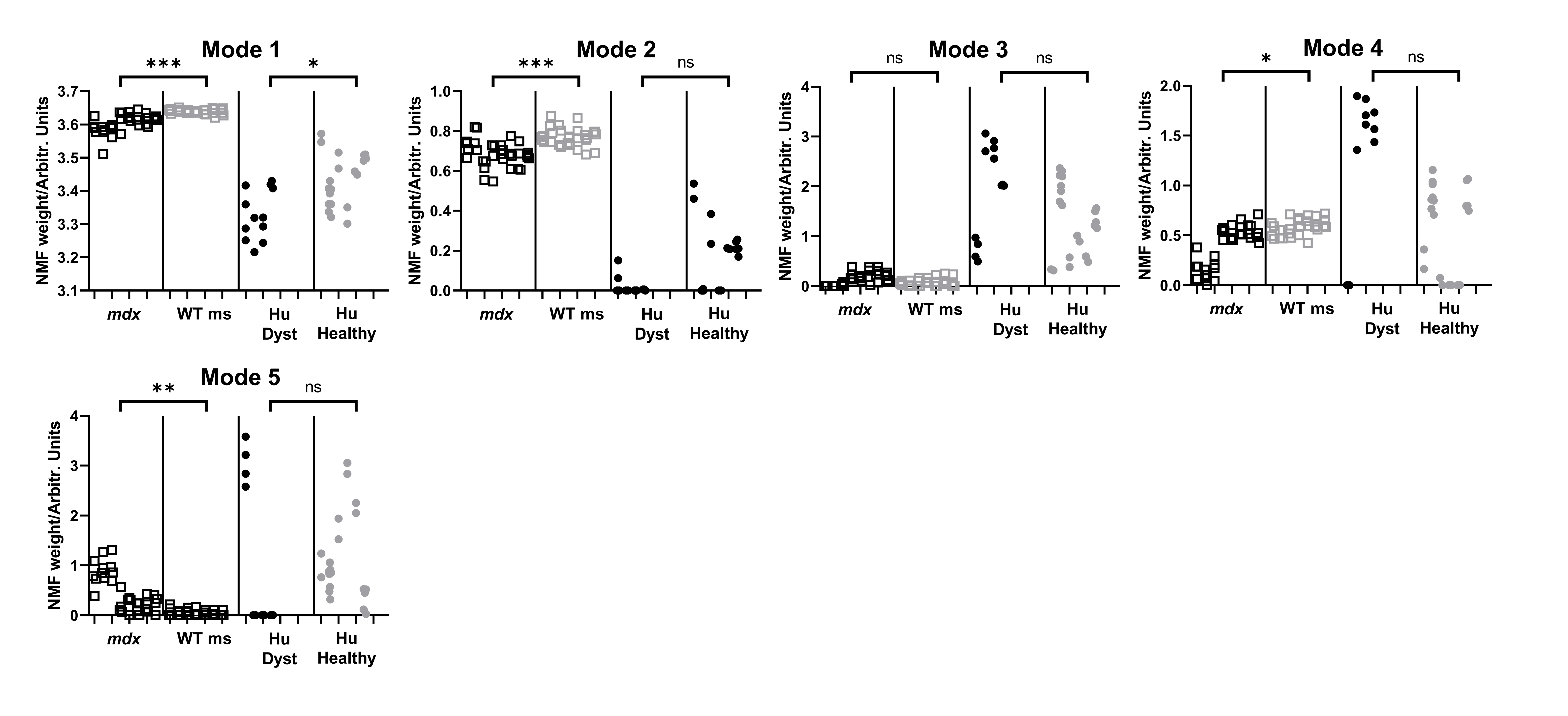

Supplement: Supplementary file 2 — Figure S2. Nested plots of NMF weight for each spectrum in the mdx/human muscular dystrophy analysis. [file JRS-54-258-s003.tiff]

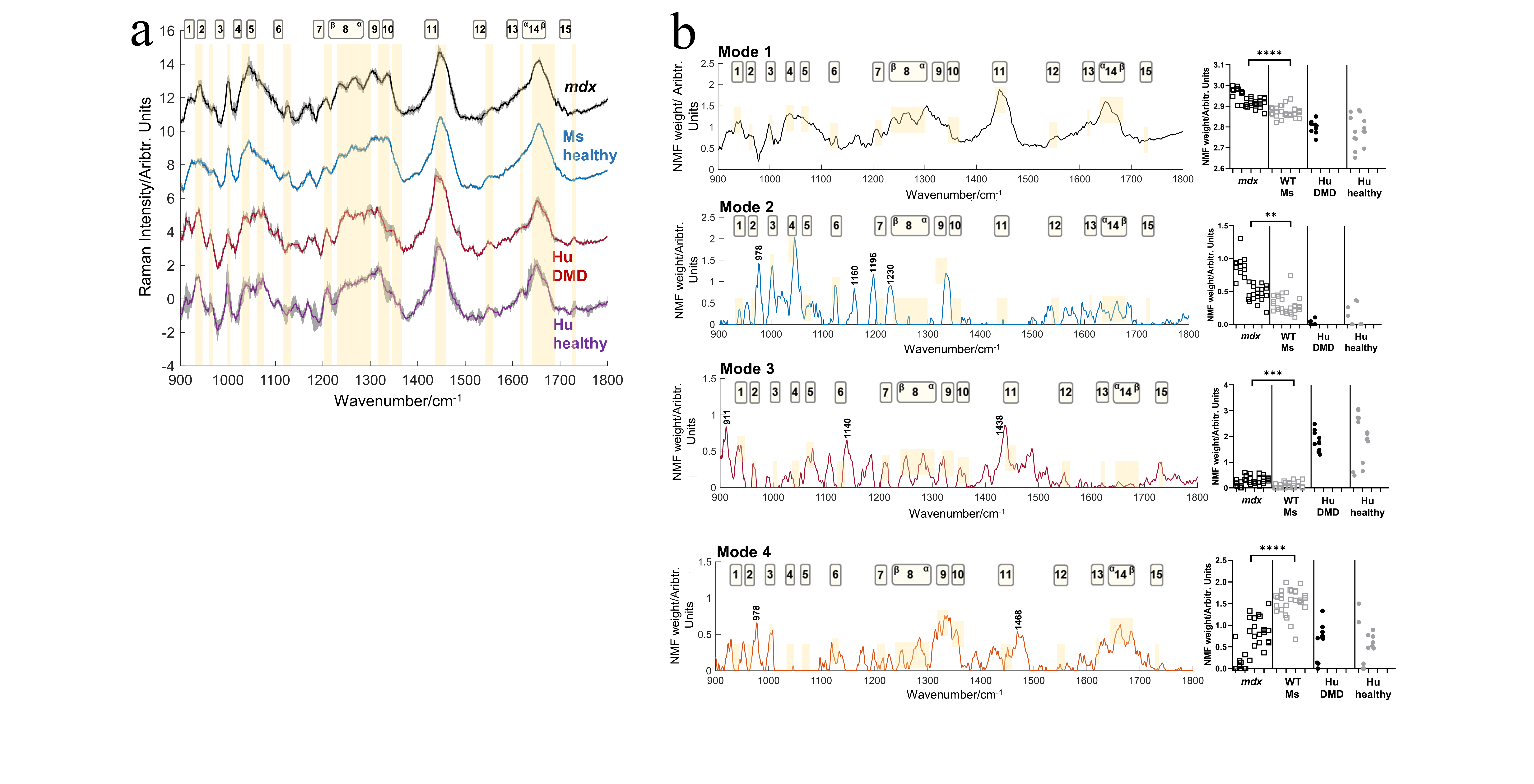

Supplement: Supplementary file 3 — Figure S3. Average spectra and NMF modes for mdx, and human DMD analyses [file JRS-54-258-s004.tiff]
