# Supplementary material for: Non‐negative matrix factorisation of Raman spectra finds common patterns relating to neuromuscular disease across differing equipment configurations, preclinical models and human tissue
Source: J Raman Spectrosc. 2022 Dec 22;54(3):258–68. doi: 10.1002/jrs.6480 (PMC10947050; doi:10.1002/jrs.6480)
Supplement: Supplementary file 4 — Table S1. Demographic and clinical details for the human participants used in the microscope/fibre optic probe study. Table S2. Demographic and clinical details for the human participants used in the mouse/human study presented in the main manuscript. Age/gender matched healthy volunteer samples were selected from those presented in supplementary Table 1. Table S3. Demographic and clinical details for the human participants used in the mouse/human study presented in the supplement data. Healthy volunteer samples were selected from those presented in supplementary Table 1. Table S4. Tentative peak assignments of prominent spectral features in the average spectra plots. Table S5. Tentative peak assignments for additional spectral features identified within the NMF mode plots. Table S6. Classification performance on individual spectra: probe/microscope – human mitochondrial disease vs. healthy volunteers. Table S7. Classification performance on individual spectra in the mdx/human muscular dystrophy analysis. Table S8. Classification performance using all modes: mdx vs. human DMD. For comparison, results achieved by PCA‐LDA using PCs which cover 90% of the data variance are shown. [file JRS-54-258-s002.docx]

|  | **Mitochondrial myopathy (n=14)** | **Healthy volunteers (n=10)** |
| --- | --- | --- |
| **Gender** Male: Female | 8:6 | 6:4 |
| **Age** Mean (range, yrs) | 51 (29-80) | 33 (17-54) |
| **Muscle biopsied** |  |  |
| Biceps | - | 4 |
| Quadriceps | 3 | 5 |
| Tibialis Anterior | 11 | - |
| Deltoid | 1 | 9 |
| Hamstrings |  | 10 |
| **Clinico-pathological Diagnoses (n)** |  |  |
| m.3243A>G variant | 11 |  |
| POLG-related mitochondrial disease | 3 |  |
| Single large-scale mtDNA deletion | 1 |  |

**Supplementary table 1. Demographic and clinical details for the human participants used in the microscope/fibre optic probe study.**

|  | **Dystrophic myopathy (n=4)** | **Healthy volunteers (n=6)** |
| --- | --- | --- |
| **Gender** Male: Female | 4:0 | 6:0 |
| **Age** Mean (range, yrs) | 47 (38-72) | 34 (17 – 52) |
| **Muscle biopsied** |  |  |
| Deltoid | 2 |  |
| Quadriceps | 2 |  |
| Hamstrings |  | 6 |
| **Clinico-pathological Diagnoses (n)** |  |  |
| Limb girdle muscular dystrophy (all genetics negative) | 3 |  |
| Desmin myopathy | 1 |  |

**Supplementary table 2. Demographic and clinical details for the human participants used in the mouse/human study presented in the main manuscript. Age/gender matched healthy volunteer samples were selected from those presented in supplementary table 1.**

|  | **Duchenne muscular dystrophy (n=2)** | **Healthy volunteers (n=4)** |
| --- | --- | --- |
| **Gender** Male: Female | 2:0 | 4:0 |
| **Age** Mean (range, yrs) | 7 (4 and 10) | 25 (17-33) |
| **Muscle biopsied** |  |  |
| Quadriceps | 2 |  |
| Hamstrings |  | 4 |

**Supplementary table 3. Demographic and clinical details for the human participants used in the mouse/human study presented in the supplement data. Healthy volunteer samples were selected from those presented in supplementary table 1.**

| **Wavenumber region** | **Wavenumbers (cm^-1^)** | **Assignment** |
| --- | --- | --- |
| 1 | 932-940 | Protein (α-helix)^[1-2]^ |
| 2 | 966 | Nucleic acids, triple helix vibrations^[3]^ |
| 3 | 998-1006 | Phenylalanine^[4]^ |
| 4 | 1035-1045 | Proteins (collagen)^[5-6]^ |
| 5 | 1065-1075 | Lipids (phospholipids, triglycerides)^[7-8]^ |
| 6 | 1120-1130 | Proteins (collagen), lipids^[7, 9]^ |
| 7 | 1205-1215 | Tyrosine, phenylalanine^[2, 6]^ |
| 8 | 1230-1300 | Proteins (amide III; *β-sheet, **α-helix)^[4, 10]^ |
| 9 | 1315-1340 | CH_2_CH_3_ deformation, proteins/lipids^[11]^ |
| 10 | 1345-1365 | Nucleic acids, tryptophan^[1, 11]^ |
| 11 | 1440-1454 | CH modes (CH_2_ and CH_3_ deformations: bending & scissoring) in proteins/lipids^[10, 12]^ |
| 12 | 1546-1556 | Tryptophan, proteins^[13-14]^ |
| 13 | 1614-1620 | C=C stetch, tryptophan, proteins^[4-5, 15]^ |
| 14 | 1645-1685 | Proteins (amide I, **α -helix, *β -sheet/unordered)^[2, 16-17]^ |
| 15 | 1730 | Lipid (ester C=O stretching)^[18]^ |

**Supplemental table 4. Tentative peak assignments of prominent spectral features in the average spectra plots.**

| **Wavenumber(s)**  **(cm^-1^)** | **Assignment** |
| --- | --- |
| 911 | Glucose^[19]^ |
| 915 | Nucleic acids^[20]^ |
| 951 | Protein (α-helix)^[21]^ |
| 978 | Phospholipids^[16]^ |
| 988 | Phenylalanine^[22]^ |
| 1107 | Nucleic acids^[23]^ |
| 1140 | Collagen^[24]^ |
| 1145 | Unassigned |
| 1160 | C-C stretching in protein^[25]^ |
| 1180/84 | Cytosine/guanine/adenine^[2, 25]^ |
| 1196 | Cytosine^[26]^ |
| 1227/1230 | β-sheet amide III^[16]^ |
| 1415 | CH rocking in lipids^[27]^ |
| 1430 | Lipids (CH_2_ scissoring)^[28]^ |
| 1436 | CH_2_ deformation protein^[29]^ |
| 1466/1468 | Guanine, adenine^[22]^ |
| 1485 | Guanine/adenine^[2]^ |
| 1496 | Protein^[22]^ |
| 1516/1520/1529 | Carotenoids^[2, 30]^ |
| 1634/1684 | Amide I^[31-32]^ |

**Supplemental table 5. Tentative peak assignments for additional spectral features identified within the NMF mode plots.**


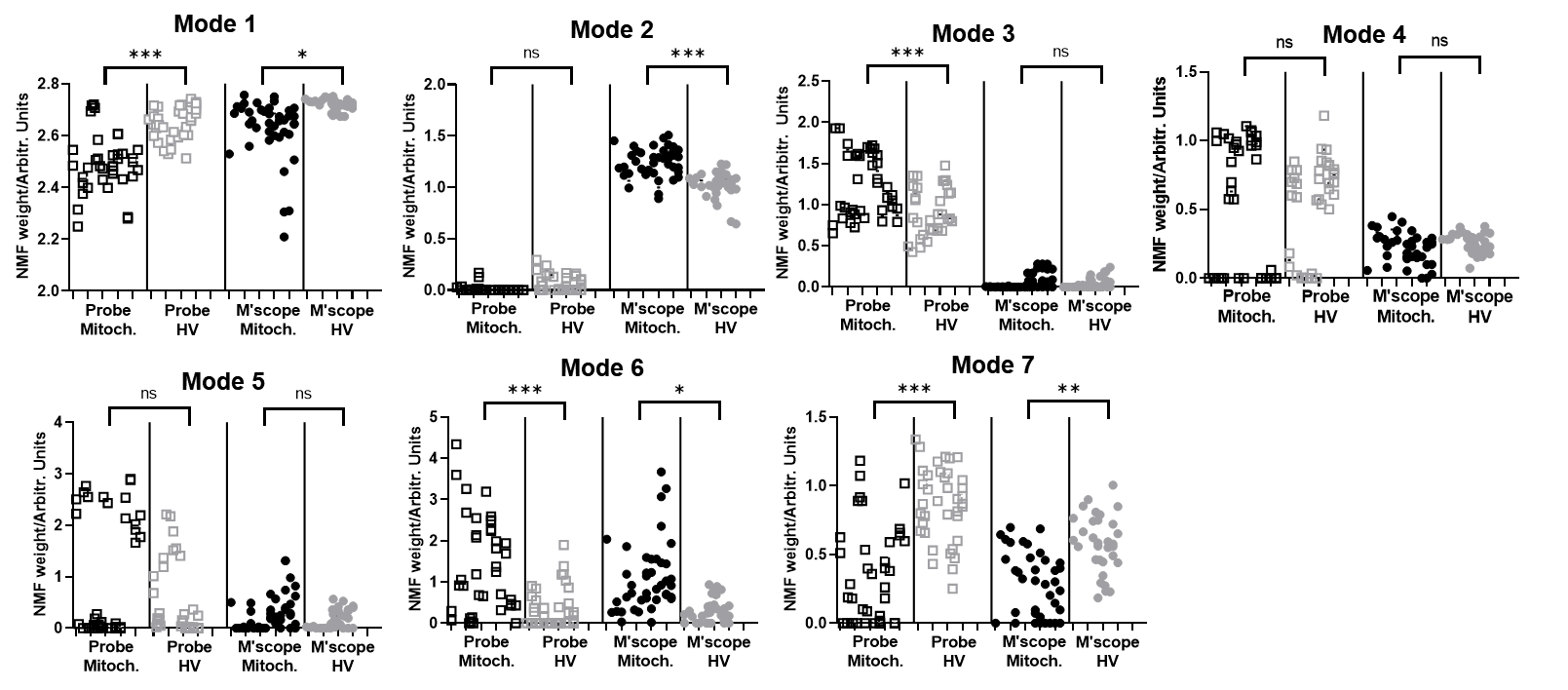


**Figure S1. Nested plots of NMF weight for each spectrum in the probe microscope analysis.**

Spectral weights are shown nested within each sample.

Modes 1, 6 and 7 demonstrated significant differences between patients and healthy volunteers in the same direction for both probe and microscope.

Modes 2 and 3 detect patterns specific to microscope and probe formats, respectively.

Mitoc. – Mitochondrial, M’scope – Microscope, HV – Healthy volunteers.

| **ALL MODES** | | | | |
| --- | --- | --- | --- | --- |
|  | **Accuracy** | **Sensitivity** | **Specificity** | **AUROC** |
| **MICROSCOPE** | | | | |
| **NMF-LDA** | 82.1% (1.1) | 86.1% (1.8) | 76.9% (1.2) | 0.88 (0.01) |
| **PCA-LDA (28 PCs)** | 60.7% (3.4) | 59.4% (4) | 62.5% (5.4) | 0.62 (0.03) |
| **PROBE** | | | | |
| **NMF-LDA** | 78.2% (0.9) | 73.2% (1.2) | 82.6% (1.4) | 0.82 (0.01) |
| **PCA-LDA (25 PCs)** | 62.9% (5.2) | 65.1% (8.5) | 61.1% (5.8) | 0.61 (0.05) |
|  | | | | |
| **MODES THAT ARE COMMON TO PROBE AND MICROSCOPE** | | | | |
| **MICROSCOPE** | | | | |
| **NMF-LDA (1,6,7)** | 73.4% (0.6) | 75.3% (0.8) | 70.8% (0.8) | 0.80 (0.01) |
| **PROBE:** | | | | |
| **NMF-LDA (1,6,7)** | 82.2% (0.9) | 79.7% (1.6) | 84.3% (0.9) | 0.85 (0.01) |

**Supplemental table 6. Classification performance on individual spectra: probe/microscope – human mitochondrial disease vs. healthy volunteers.**

In this cross validation, repeated leave-p(sample)-out is performed and the individual spectra are classified. Mean (standard deviation) are shown. For comparison, results achieved by PCA-LDA are also shown.


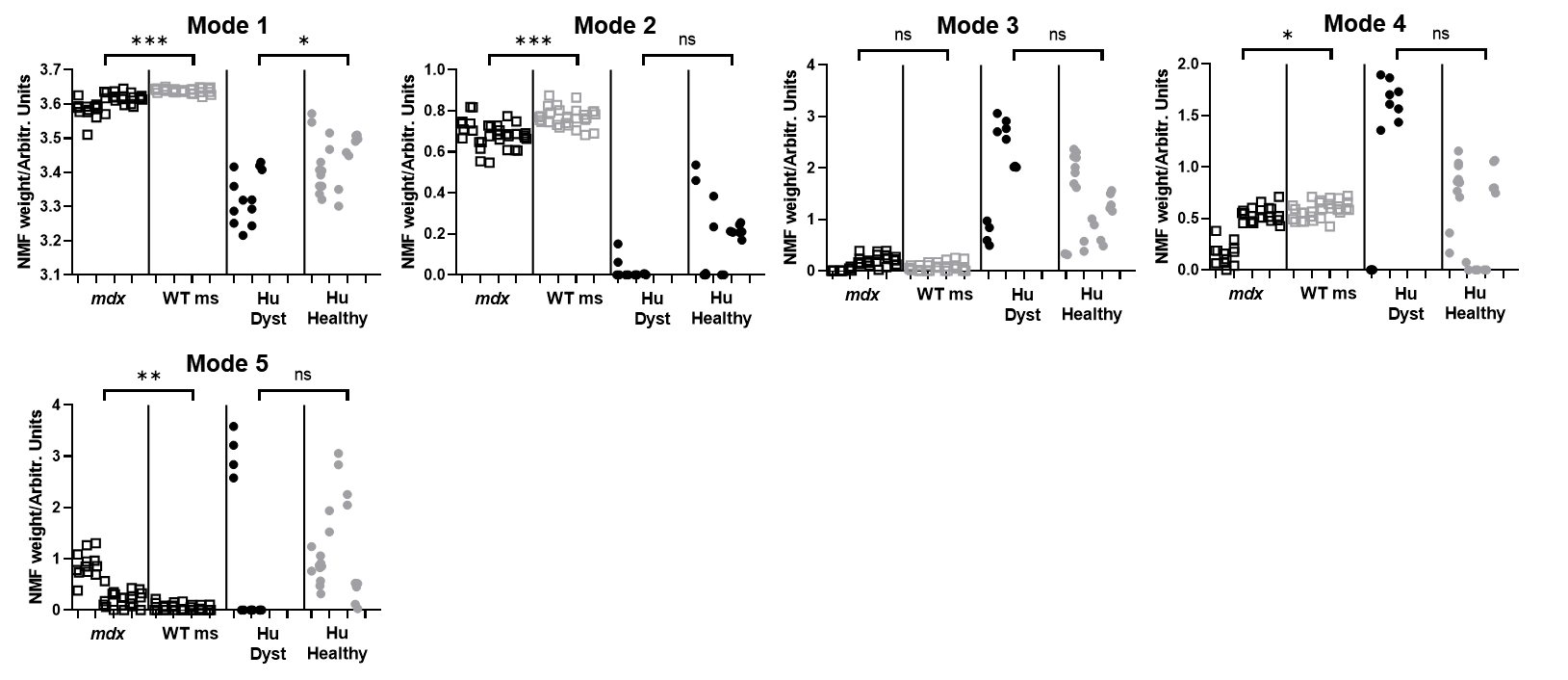


**Figure S2. Nested plots of NMF weight for each spectrum in the *mdx/*human muscular dystrophy analysis.**

Spectral weights are shown nested within each sample.

Mode 1 is significantly different in both the mouse and human comparisons in the same direction.

Modes 2 is significantly different for mdx with the human samples trending in the same direction but not reaching statistical significance.

|  | **Accuracy** | **Sensitivity** | **Specificity** | **AUROC** |
| --- | --- | --- | --- | --- |
| **USE ALL MODES** | | | | |
| **NMF-LDA** | 79.5% (3.7) | 75% (5.1) | 84.5% (4.5) | 0.89 (0.04) |
| **PCA-LDA (38 PCs)** | 80.1% (4.7) | 74.3% (7.7) | 86.7% (4.8) | 0.84 (0.05) |
|  | | | | |
| **MODES THAT ARE COMMON TO MOUSE AND HUMAN** | | | | |
| **NMF-LDA (1,2)** | 84.4% (1.8) | 79.8% (2.4) | 89.7% (2.1) | 0.91 (0.02) |

**Supplemental table 7. Classification performance on individual spectra in the *mdx*/human muscular dystrophy analysis.**

In this cross validation, repeated leave-p(mice)-out is performed and the individual spectra are classified. Mean (standard deviation) are shown. For comparison, results achieved by PCA-LDA are also shown.

**
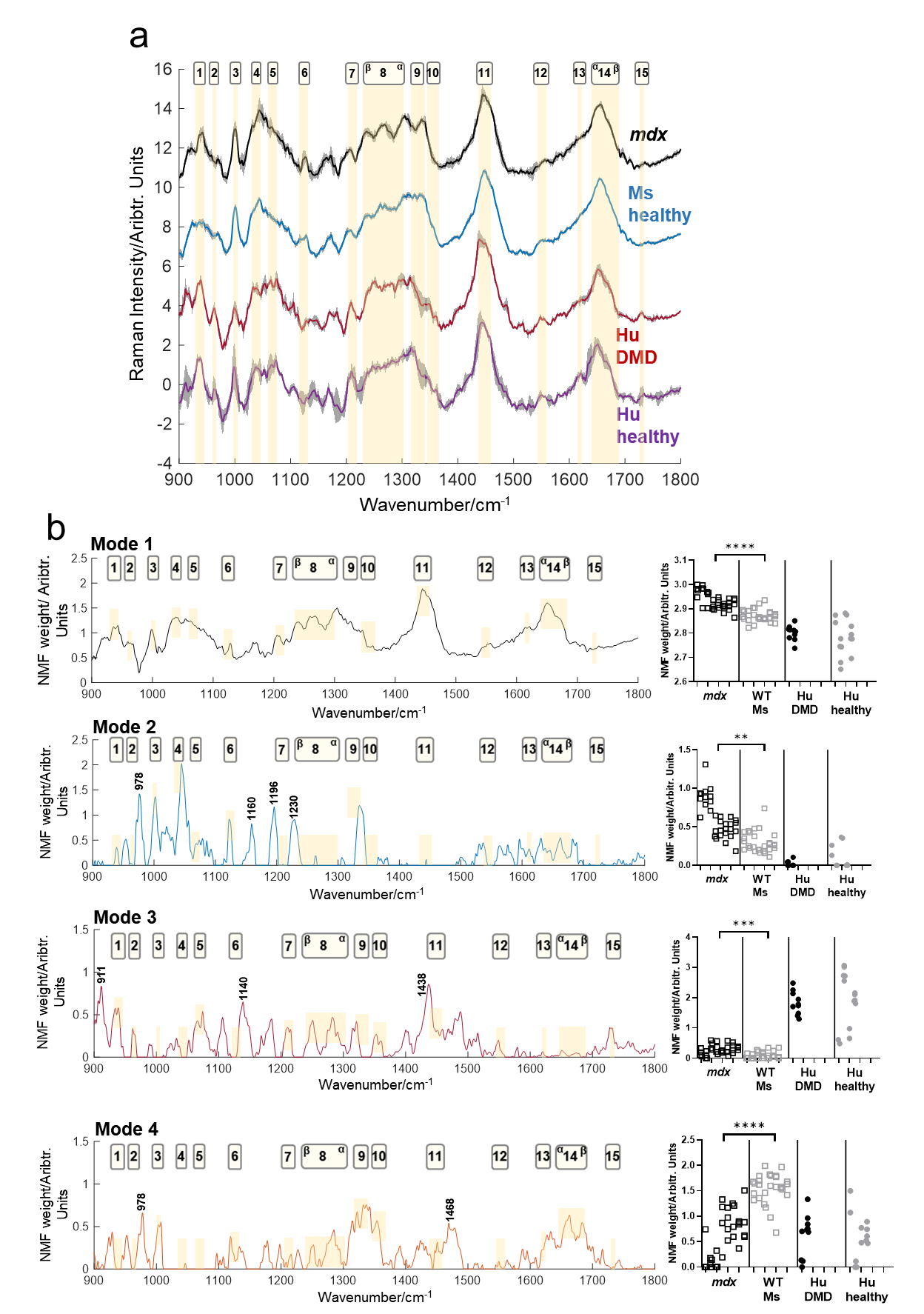
**

**Figure S3. Average spectra and NMF modes for mdx, and human DMD analyses**

Only two DMD samples were available and thus statistical analyses are limited with the human samples.

All modes show significant differences for the mice. There are no statistically significant trends for the human data, possibly as there are insufficient spectra within the factorisation to influence the modes output by the NMF.

|  | **Accuracy** | **Sensitivity** | **Specificity** | **AUROC** |
| --- | --- | --- | --- | --- |
| **Classify individual spectra (leave 3 out)** | | | | |
| **NMF-LDA** | 85.5% (1.8) | 81.6% (2.4 | 90% (2.3) | 0.94 (0.02) |
| **PCA-LDA (38 PCs)** | 79.9% (4.7) | 73.8% (7.9) | 86.8 (4.8) | 0.84 (0.06) |
| **Av modes or PCs and classify individual mice (leave 3 out)** | | | | |
| **NMF-LDA** | 98.3% (3.7) | 96.9% (0.03) | 100% (0) | 0.99 (0.03) |
| **PCA-LDA (15 PCs)** | 90.9% (5.6) | 83.1% (10.4) | 99.9% (1.3) | 0.91 (0.05) |

**Supplemental table 8. Classification performance using all modes: *mdx* vs. human DMD.** For comparison, results achieved by PCA-LDA using PCs which cover 90% of the data variance are shown.

**References**

[1] F. Bonnier and H. J. Byrne, *Analyst* **2012**, *137*, 322.

[2] N. Stone, C. Kendall, J. Smith, P. Crow and H. Barr, *Faraday Discuss.* **2004**, *126*, 141.

[3] S. Fendel and B. Schrader, *Fresenius' Journal of Analytical Chemistry* **1998**, *360*, 609.

[4] C. J. Kirkby, J. Gala de Pablo, E. Tinkler-Hundal, H. M. Wood, S. D. Evans and N. P. West, *Analyst* **2021**, *146*, 581.

[5] G. Zhu, X. Zhu, Q. Fan and X. Wan, *Spectrochim. Acta A Mol. Biomol. Spectrosc.* **2011**, *78*, 1187.

[6] J. De Gelder, K. De Gussem, P. Vandenabeele and L. Moens, *J Raman Spectrosc.* **2007**, *38*, 1133.

[7] N. Huang, M. Short, J. Zhao, H. Wang, H. Lui, M. Korbelik and H. Zeng, *Opt. Express* **2011**, *19*, 22892.

[8] A. C. S. Talari, Z. Movasaghi, S. Rehman and I. u. Rehman, *Appl. Spectrosc. Rev.* **2015**, *50*, 46.

[9] Z. Huang, A. McWilliams, H. Lui, D. I. McLean, S. Lam and H. Zeng, *Int. J. Cancer* **2003**, *107*, 1047.

[10] N. Stone, C. Kendall, N. Shepherd, P. Crow and H. Barr, *J Raman Spectrosc.* **2002**, *33*, 564.

[11] A. J. Ruiz-Chica, M. A. Medina, F. Sánchez-Jiménez and F. J. Ramírez, *J Raman Spectrosc.* **2004**, *35*, 93.

[12] L. Silveira, Jr., F. L. Silveira, B. Bodanese, R. A. Zangaro and M. T. Pacheco, *J Biomed. Opt.* **2012**, *17*, 077003.

[13] R. Gautam, S. Vanga, A. Madan, N. Gayathri, U. Nongthomba and S. Umapathy, *Anal. Chem.* **2015**, *87*, 2187.

[14] E. B. Carew, I. M. Asher and H. E. Stanley, *Science* **1975**, *188*, 933.

[15] Y. Chen, J. Dai, X. Zhou, Y. Liu, W. Zhang and G. Peng, *PLoS One* **2014**, *9*, e93906.

[16] A. Mahadevan-Jansen and R. R. Richards-Kortum, *J Biomed. Opt.* **1996**, *1*, 31.

[17] A. Mahadevan-Jansen, M. F. Mitchell, N. Ramanujam, A. Malpica, S. Thomsen, U. Utzinger and R. Richards-Kortum, *Photochem. Photobiol.* **1998**, *68*, 123.

[18] K. Czamara, K. Majzner, M. Z. Pacia, K. Kochan, A. Kaczor and M. Baranska, *J Raman Spectrosc.* **2015**, *46*, 4.

[19] J. W. Kang, Y. S. Park, H. Chang, W. Lee, S. P. Singh, W. Choi, L. H. Galindo, R. R. Dasari, S. H. Nam, J. Park and P. T. C. So, *Sci. Adv.* **2020**, *6*, eaay5206.

[20] A. Gualerzi, S. Niada, C. Giannasi, S. Picciolini, C. Morasso, R. Vanna, V. Rossella, M. Masserini, M. Bedoni, F. Ciceri, M. E. Bernardo, A. T. Brini and F. Gramatica, *Sci. Rep.* **2017**, *7*, 9820.

[21] R. Jyothi Lakshmi, V. B. Kartha, C. Murali Krishna, R. S. JG, G. Ullas and P. Uma Devi, *Radiat. Res.* **2002**, *157*, 175.

[22] K. W. Short, S. Carpenter, J. P. Freyer and J. R. Mourant, *Biophysical Journal* **2005**, *88*, 4274.

[23] L. Wang, Z. Zhang, L. Huang, W. Li, Q. Lu, M. Wen, T. Guo, J. Fan, X. Wang, X. Zhang, J. Fang, X. Yan, Y. Ni and X. Li, *Analyst* **2014**, *139*, 455.

[24] C. Yorucu, K. Lau, S. Mittar, N. H. Green, A. Raza, I. U. Rehman and S. MacNeil, *Appl. Spectrosc. Rev.* **2016**, *51*, 243.

[25] P. R. T. Jess, D. D. W. Smith, M. Mazilu, K. Dholakia, A. C. Riches and C. S. Herrington, *Int. J Cancer* **2007**, *121*, 2723.

[26] G. Pezzotti, *J Raman Spectrosc.* **2021**, *52*, 2348.

[27] H. J. Koster, T. Rojalin, A. Powell, D. Pham, R. R. Mizenko, A. C. Birkeland and R. P. Carney, *Nanoscale* **2021**, *13*, 14760.

[28] N. Kuhar, S. Sil, T. Verma and S. Umapathy, *RSC Advances* **2018**, *8*, 25888.

[29] N. Amharref, A. Beljebbar, S. Dukic, L. Venteo, L. Schneider, M. Pluot and M. Manfait, *Biochim. Biophys. Acta* **2007**, *1768*, 2605.

[30] B. Brozek-Pluska, K. Miazek, J. Musiał and R. Kordek, *RSC Advances* **2019**, *9*, 40445.

[31] G. Sieler and R. Schweitzer-Stenner, *J Am. Chem. Soc.* **1997**, *119*, 1720.

[32] M. Khalid, T. Bora, A. A. Ghaithi, S. Thukral and J. Dutta, *Sci. Rep.* **2018**, *8*, 9417.
